# Game theoretical mapping of white matter contributions to visuospatial attention in stroke patients with hemineglect

**DOI:** 10.1002/hbm.24987

**Published:** 2020-04-03

**Authors:** Monica N. Toba, Melissa Zavaglia, Caroline Malherbe, Tristan Moreau, Federica Rastelli, Anna Kaglik, Romain Valabrègue, Pascale Pradat‐Diehl, Claus C. Hilgetag, Antoni Valero‐Cabré

**Affiliations:** ^1^ Cerebral Dynamics, Plasticity and Rehabilitation Team, Frontlab, Paris Brain Institute, ICM, Sorbonne Universités, UPMC Paris 06, Inserm UMR S 1127, CNRS UMR 7225, F‐75013, & IHU‐A‐ICM Paris France; ^2^ Institute of Computational Neuroscience, University Medical Center Hamburg‐Eppendorf Hamburg Germany; ^3^ AP‐HP, HxU Pitié‐Salpêtrière‐Charles‐Foix, service de Médecine Physique et de Réadaptation & PHRC Régional NEGLECT Paris France; ^4^ Laboratory of Functional Neurosciences (EA 4559) University of Picardie Jules Verne Amiens France; ^5^ Jacobs University Focus Area Health Bremen Germany; ^6^ Centre for NeuroImaging Research—CENIR, Paris Brain Institute, ICM, Sorbonne Universités, Inserm UMR S 1127, CNRS UMR 7225, F‐75013 Paris France; ^7^ GRC‐UPMC n° 18‐Handicap cognitif et réadaptation Paris France; ^8^ Department of Health Sciences Boston University 635 Commonwealth Ave. Boston Massachusetts 02215 USA; ^9^ Laboratory for Cerebral Dynamics, Plasticity & Rehabilitation Boston University School of Medicine Boston Massachusetts 02118 USA; ^10^ Department of Neurology, Head and Neuro Center University Medical Center Hamburg‐Eppendorf Hamburg Germany

**Keywords:** brain‐behavior relationships, clinical anatomical correlations, disconnection, game theory, lesion inference, multiperturbation Shapley value analysis (MSA), neglect, stroke, visuospatial attention, white matter

## Abstract

White matter bundles linking gray matter nodes are key anatomical players to fully characterize associations between brain systems and cognitive functions. Here we used a multivariate lesion inference approach grounded in coalitional game theory (multiperturbation Shapley value analysis, MSA) to infer causal contributions of white matter bundles to visuospatial orienting of attention. Our work is based on the characterization of the lesion patterns of 25 right hemisphere stroke patients and the causal analysis of their impact on three neuropsychological tasks: *line bisection*, *letter cancellation*, and *bells cancellation*. We report that, out of the 11 white matter bundles included in our MSA coalitions, the optic radiations, the inferior fronto‐occipital fasciculus and the anterior cingulum were the only tracts to display task‐invariant contributions (positive, positive, and negative, respectively) to the tasks. We also report task‐dependent influences for the branches of the superior longitudinal fasciculus and the posterior cingulum. By extending prior findings to white matter tracts linking key gray matter nodes, we further characterize from a network perspective the anatomical basis of visual and attentional orienting processes. The knowledge about interactions patterns mediated by white matter tracts linking cortical nodes of attention orienting networks, consolidated by further studies, may help develop and customize brain stimulation approaches for the rehabilitation of visuospatial neglect.

## Graphical abstract

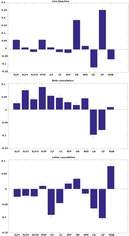

We inferred causal contributions of white matter bundles to visuo‐spatial attention. We observed that the optic radiations and the inferior fronto‐occipital fasciculus exerted task‐invariant positive contributions to spatial attention, whereas the anterior cingulum provided task‐invariant negative ‐hindering‐ contributions. The superior longitudinal fasciculus and the corpus callosum displayed task‐dependent contributions. These results may inform future uses of brain stimulation approaches in visuospatial attention rehabilitation.

## 1 INTRODUCTION

Conceptualizations concerning the fundamental organization of the human brain have considered cognitive processes as emerging from extended neural networks made of specialized gray matter areas wired by white matter tracts (Von Bonin & Bailey, 1947). With this same framework in mind, Geschwind (1965) rescued the notion of “disconnection” syndrome, and defined this type of injuries as neurological deficits emerging from interrupted white matter bundles. Since then, the use of whole brain neuroimaging techniques and the development of multivariate analytic approaches have significantly extended the structural and functional characterization of distributed network models.

Gray matter damage often causes the loss of specialized brain functions. Alone or combined with the former, white matter dysfunctions by either insufficient (i.e., what is popularly referred to as disconnection syndromes) or excessive connectivity, have been hypothesized as the cause of even more severe cognitive impairments (Catani & Ffytche, 2005). White matter injuries can be generated through direct damage to tracts and bundles. Pure forms are however rare to find in clinical settings, and occur most often by the Wallerian degeneration of axonal projections attached to affected neuronal bodies, as a consequence of cortical lesions (Catani & Ffytche, 2005). Highlighting the importance of white matter dysfunction (often characterized as disconnection syndromes), it has been well documented that for injuries of equal volume, white matter damage can be much more impairing than gray matter lesions (Corbetta et al., 2015; Doricchi, Thiebaut de Schotten, Tomaiuolo, & Bartolomeo, 2008). This is because the former can impact the flow of information throughout extended systems of interacting brain nodes (Catani & Ffytche, 2005), disrupting the function of multiple cortical areas simultaneously (Bartolomeo, 2007). Additionally, if under certain circumstances focal gray matter damage may be limited by compensatory plasticity, white matter injuries impact a whole network of interconnected areas, limiting the efficacy of functional remapping by cortical or subcortical vicariant systems (see, e.g., Catani & Mesulam, 2008; Doricchi et al., 2008; Duffau, 2005). However, in the domain of neurological rehabilitation, the significance of white matter tract integrity (hence some instances, the impact of disconnection syndromes) on human cognition and behaviors demands careful and detailed characterization.

Different technological approaches have significantly contributed to build a framework from which to investigate the functional roles of white matter tracts and model the impact of disconnection phenomena. The most relevant of all has been diffusion neuroimaging, which opened the door to in vivo studies of white matter bundles in the human brain (Basser, Mattiello, & LeBihan, 1994). Thanks to this technology, tractography by diffusion‐tensor imaging (DTI) has now become a widely used method for the “virtual dissection” and characterization of white matter connectivity in healthy participants or neuropsychiatric patients (Basser, Pajevic, Pierpaoli, Duda, & Aldroubi, 2000; Le Bihan et al., 2001). Most recently, reliable probabilistic atlases of human white matter tracts developed with DTI, used in conjunction with structural T1 MRI sequences and lesion masks allow the identification and quantification of altered bundles without the need of diffusion datasets (Foulon et al., 2018). Finally, innovative mathematical models integrating alterations of neurophysiological (electroencephalography [EEG] and magnetoencephalography [MEG]) or hemodynamic (functional MRI [fMRI]) datasets from specific gray matter regions, and behavioral/clinical information have proven instrumental to unearth causal evidence supporting interactions among brain areas and their functional links. However, the features of “disconnection syndromes” addressed with all these approaches are considered for many conceptually different from exploring events of the original notion defined by Geschwind (1965), hence, they must only be interpreted cautiously as part of the latter framework.

In a recent study (Toba et al., 2017), we used a computational approach based on multiperturbation Shapley value analysis (MSA) (Keinan, Sandbank, Hilgetag, Meilijson, & Ruppin, 2004; see also Kaufman, Serfaty, Deouell, Ruppin, & Soroker, 2009, for an application of this method in stroke patients with neglect) to identify in stroke datasets gray matter nodes with causal bearing on attentional orienting. To this end, we integrated individual lesion maps and neuropsychological scores from stroke patients diagnosed with hemispatial neglect and explored causal regional contributions to the outcomes of three neuropsychological visuospatial tasks used to diagnose the severity of this symptom (Toba et al., 2017). Our results identified significant gray matter contributors such as the intraparietal sulcus (IPS), the temporoparietal junction (TPJ), and the inferior frontal gyrus (IFG).

Most importantly, our analyses predicted synergistic interactions between these areas, corresponding to key white matter tracts such as the branches of the superior longitudinal fasciculus (SLF). The latter system is the main component of a right lateralized frontoparietal attentional network involved in *endogenous* and *exogenous* attentional orienting, also contributing to the modulation of conscious visual performance (Bartolomeo, Thiebaut de Schotten, & Doricchi, 2007; Chica et al., 2012; Corbetta & Shulman, 2002; Thiebaut de Schotten et al., 2005; Chanes, Chica, Quentin, & Valero‐Cabre, 2012; Chica, Bartolomeo, & Valero‐Cabre, 2011; Quentin, Chanes, Vernet, & Valero‐Cabre, 2015; Quentin et al., 2016). Compatible with prior work on white matter disconnections (see pioneer work by Critchley (1953), Geschwind (1965), and Mesulam (1981)), this first study opened new avenues for the causal characterization of interregional influences, subserving specific behaviors, placing the focus on the role played by functional interactions rather than on cortical nodes (Toba et al., 2017).

In the current study, we adopted this same MSA approach and modeled this time the causal impact of white matter disconnections in the same cohort of right hemisphere stroke patients on which gray matter contributions had been previously characterized (Toba et al., 2017). To this end, we relied on a probabilistic white matter atlas (Foulon et al., 2018) and using lesion masks outlined on T1‐MRI sequences, we characterized a correlate of white matter dysfunction by estimating the percentage of tract disconnection for each individual patient and bundle of interest. We then studied potential interactions between white matter tracts identified as likely contributors to visuospatial orienting of attention. More specifically, we first considered for our study the three branches of the SLF mentioned above (branches connecting the gray‐matter regions used as players in a prior MSA study in the gray matter; Toba et al., 2017). Second, based on a priori hypothesis issued from lesion studies conducted in neurological patients with visuospatial attention deficits and neglect, the following candidate tracts were also initially considered for our coalitions: (a) the inferior fronto‐occipital fasciculus (IFOF) connecting the ventrolateral prefrontal cortex and the medial orbitofrontal cortex with the occipital lobe (Urbanski et al., 2008; Vaessen, Saj, Lovblad, Gschwind, & Vuilleumier, 2016); (b) the inferior longitudinal fasciculus (ILF), linking temporal and occipital visual areas to the amygdala and the hippocampus (Bird et al., 2006); and (c) the interhemispheric commissural projections of the corpus callosum (CC) (Lunven et al., 2015, see also Bozzali et al., 2012 and Vaessen et al., 2016). Third, preliminary analyses conducted with the white matter tracts mentioned above showed that other bundles and not just those considered in our analyses also contributed to the tasks. Consequently, we included in subsequent analyses: (a) the posterior segment of the arcuate fasciculus (APS), connecting Wernicke's region, the inferior parietal lobe and the superior and middle temporal gyri (Urbanski et al., 2011); (b) the anterior thalamic projections (ATP) traveling from the thalamus to attentional cortical regions (Hattori et al., 2017); (c) the anterior cingulum (CA) and the posterior cingulum (CP), linking the uncus and the parahippocampal gyrus to subgenual portions of the orbitofrontal cortex and the cingulate cortex, the superior medial frontal gyrus, the paracentral lobule to the precuneus, cuneus, lingual, and fusiform gyri (Catani, Dell'acqua, & Thiebaut de Schotten, 2013; Crosby, Humphrey, & Lauer, 1962; Husain & Schott, 2016; Nieuwenhuys, Voogd, & van Huijzen, 2008); and (d) the optic radiations (OR), linking the lateral geniculate nucleus to the primary visual cortex.

By using a multivariate lesion inference approach based on MSA (Keinan et al., 2004), we identified positive and negative contributions of such tracts to visuospatial performance outcomes. A negative MSA contribution denoted that a white matter tract dysfunction resulted in paradoxical performance improvements, whereas the same effects on a white matter tract exerting positive contribution would generate performance decreases.

## 2 MATERIALS AND METHODS

### 2.1 Patient recruitment demographics and consent form

In the present study, we analyzed existing MRI and behavioral datasets of a cohort of 25 right‐handed stroke patients (17 men, mean age 55.96 years, *SD* 10.63, range 35–79) previously explored with different goals (Toba et al., 2017), evaluated by the PHRC Regional NEGLECT trial (a multicentric double blind clinical trial evaluating efficacy and safety of a repetitive transcranial magnetic stimulation [rTMS] regime for 2 weeks to improve visuospatial neglect in chronic stroke patients, with a 6 months follow‐up), and databased by the Centre for Cognitive Anatomy (CAC) at the Paris Brain Institute, Pitié‐Salpêtrière Hospital, Paris, France. Patients presented for the first time a stroke in the right hemisphere and were neuropsychologically evaluated at least 2 months following the stroke event after verifying stability of neglect symptoms in two consecutive measures. Twenty patients were clearly at the chronic stage, well beyond 3 months poststroke, at the time they were last evaluated for our study. The remaining five patients were recruited following the stated criteria but evaluated 2–3 months poststroke, hence in the transition between the “subacute” and “chronic” stage, after verifying that they showed stable neglect deficits (see details in Table 1). The mean period poststroke onset at the time of testing was ~5 months poststroke (212.48 ± 269.01 days, range 64–1,434 days). The visual field of all the patients was assessed first clinically by an expert neurologist and in a majority of patients with an ad hoc visual perimetry test performed during the days following the last evaluation at the neuro‐ophthalmology department at the Pitié‐Salpêtrière Hospital. All patients provided informed consent according to the local ethical committee regulations (CCP Ile de France IV/Ile de France I). For details on the demographic and clinical characteristics of the same study refer to Table 1 and previously published work (Toba et al., 2017).

**Table 1:** Demographic and clinical characteristics of the 25 patients included in the analyses

Patient number	Sex and age	Illness onset (days)	Stroke etiology	Visual field deficits	Line bisection (% deviation)	Bells cancellation (left/right found target, max 15/15)	Letter cancellation (left/right found targets, max 30/30)
1	M, 59	452	Ischemic	Left extinction	+3.8	14/15	28/29
2	M, 43	81	Ischemic	Left extinction	+18.2	11/10	–
3	F, 62	227	Ischemic	Normal	−5.6	8/11	19/29
4	M, 55	95	Ischemic	Left extinction	+2.72	12/11	29/29
5	M, 61	83	Ischemic	Left extinction	−4.8	14/11	29/21
6	F, 35	118	Hemorrhagic	Normal	−0.4	15/15	21/23
7	M, 53	64	Ischemic	Normal	–	15/15	–
8	M, 57	82	Ischemic	Normal	−1.8	15/10	29/30
9	M, 41	308	Hemorrhagic	Left hemianopia	+10.4	11/13	30/30
10	M, 37	142	Hemorrhagic	Left hemianopia	+63.6	–	–
11	M, 46	209	Ischemic	Normal	+1	15/15	–
12	F, 68	208	Ischemic	Normal	–	15/15	–
13	F, 66	207	Ischemic	Left extinction	+8.2	7/14	6/24
14	M, 66	137	Ischemic	Normal	−6	12/15	27/28
15	M, 66	74	Hemorrhagic	Normal	+4.7	15/13	–
16	M, 58	187	Hemorrhagic	Normal	+0.8	14/13	20/24
17	F, 60	103	Hemorrhagic	Left hemianopia	+7.9	14/15	25/29
18	M, 57	228	Hemorrhagic	Left hemianopia	+19.5	3/13	17/27
19	F, 62	194	Hemorrhagic	Left extinction	+2.8	12/15	10/30
20	F, 49	150	Hemorrhagic	Left hemianopia	+38.2	0/13	6/29
21	M, 44	1,434	Ischemic	Left hemianopia	+20	12/13	27/27
22	M, 56	202	Ischemic	Left extinction	+6.1	14/13	30/30
23	M, 66	151	Ischemic	Left extinction	−7.4	13/13	29/30
24	F, 79	98	Ischemic	Normal	+3.9	11/12	30/27
25	M, 53	78	Ischemic	Normal	+0.4	14/14	28/30

*Note:* Positive values indicate rightward shift; negative values indicate leftward shift. “–” indicate missing data.

### 2.2 Neuropsychological evaluation tests

Neuropsychological evaluations included at least one of the following three neuropsychological tests: (a) the *line bisection test* (Schenkenberg, Bradford, & Ajax, 1980), (b) the *bells cancellation test* (Gauthier, Dehaut, & Joanette, 1989), and (c) the *letter cancellation test* (Mesulam, 1985). For each test, the representative scores and cut‐off values were computed according to well‐established guidelines (Mesulam, 1985; Rousseaux et al., 2001; Toba et al., 2017 for details). Generally, the evaluation of each patient included all three tests. Nonetheless, several of them either refused or given their state of fatigue, felt eventually unable to perform some of the three tests. Twenty‐three out of 25 patients completed the line bisection test, which measured the deviation of the midpoint labeled by the patient with regard to the exact middle of 20 cm‐long lines (Azouvi et al., 2006; Rousseaux et al., 2001). Scores larger than +6.5 mm (positive numbers indicating a rightward deviation) or shorter than −7.3 mm (negative numbers indicating a leftward deviation) were considered pathological (Rousseaux et al., 2001). Patients were asked to consecutively bisect five lines (Table 1 presents the average percent deviation across the five lines, relative to the total length of the line, i.e., 20 cm). Twenty‐four out of 25 patients completed the *bells cancellation* test which was assessed by computing the difference between the bells canceled (i.e., consciously identified by crossing or circling them out) on the right side (maximum of 15) of the sheet relative to the left side (maximum of 15). Scores larger than 2 in absolute value were considered pathological (Rousseaux et al., 2001). The *letter cancellation* test (Mesulam, 1985) was completed in 19 out of 25 patients. In this test, we estimated a laterality score calculated as the number of omitted “A” targets (i.e., targets not canceled or consciously identified by circling them out) on the right side, relative to the number omitted on left side of the page. For ages above 50 and equal to 80 years old, the omission of one target in each hemifield was considered normal (Mesulam, 1985). Consequently, scores larger than two in absolute value were considered pathological.

### 2.3 Selection of regions of interest

In our previous MSA study (Toba et al., 2017), five gray matter regions of interest (ROI) were selected in order to compose the coalition of players and conduct MSA analyses. The choice of such five ROIs was hypothesis‐driven and based on published fMRI and TMS evidence from healthy participants showing their involvement in visuospatial attention. The five ROIs comprised: the frontal eye fields (FEF), the IPS, the IFG, the TPJ and the inferior occipital gyrus (IOG). More specifically, the FEF was included in our coalition on the basis of fMRI data identifying this region as part of the dorsal attentional orienting network (Corbetta, Patel, & Shulman, 2008), and causal TMS evidence highlighting its role enhancing conscious detection of near‐threshold visual stimuli (Chanes et al., 2012; Chanes, Quentin, Tallon‐Baudry, & Valero‐Cabre, 2013; Chica, Valero‐Cabre, Paz‐Alonso, & Bartolomeo, 2014; Quentin et al., 2015; Quentin et al., 2016; see Vernet, Quentin, Chanes, Mitsumasu, & Valero‐Cabre, 2014 for a review). The inclusion of the IPS as a coalition player was based on fMRI evidence (Kincade, Abrams, Astafiev, Shulman, & Corbetta, 2005), supporting activations of this region when participants oriented their attention endogenously, and additionally TMS data emphasizing a causal role for this region in both endogenous and exogenous attentional orienting (Chica et al., 2011). Further, TPJ was also integrated in the coalition given fMRI evidence supporting its role as a key node of the ventral attentional network (Corbetta et al., 2008) engaged for the exogenous reorienting of attention, only when stimuli are behaviorally relevant for the task at hand (Chica et al., 2011; Kincade et al., 2005). The inclusion of the IFG in the coalition of our prior MSA study was justified on the basis of its role, as part of the ventral attentional network, in the reorienting of attention to unexpected task‐relevant events (Corbetta et al., 2008) and contributions to exogenous shifts of attention (Kincade et al., 2005). Finally, the IOG was also included in the coalition of gray matter ROIs considering its role as a key node of circuits comprising regions of the extrastriate visual cortex that may mark a location (Kincade et al., 2005) and contribute to attentional orienting paradigms such as line bisection judgment requiring the estimation of horizontal lengths (Fink, Marshall, Weiss, Toni, & Zilles, 2002; Waberski et al., 2008).

For the present study, we first selected a core of white matter bundles, specifically, the three branches of the SLF (known as SLF I, II, and III) from dorsal to ventral, known to physically bind some of the gray matter ROIs (i.e., FEF, IPS, IFG, and TPJ) tested in our prior study (Toba et al., 2017). Additionally, several bundles which have been consistently reported in lesion studies as involved in the orienting of spatial attention, were added to that list, which included in the end: the three branches of the superior longitudinal fasciculus (SLF I, SLF II, SLF III), the IFOF (Urbanski et al., 2008; Vaessen et al., 2016), the ILF (Bird et al., 2006), and the CC (Lunven et al., 2015, see also Bozzali et al., 2012 and Vaessen et al., 2016). Preliminary MSA analyses conducted on our preselected bundles allowed us to assess the validity of the white matter selection. We observed that several right hemisphere white matter tracts not initially included in our coalition and used for further MSA analyses were also involved in the outcomes of some of the tested neuropsychological tasks. Consequently, we opted for adding to our analyses the ATP, the OR, the APS, the CA and the CP. In sum, the rationale behind the a priori selection of a coalition of players practiced in the present study was based on empirical data issued from hypothesis‐driven approaches based on: (a) Prior fMRI and TMS literature (which guided the selection of ROI representing projection ROIs of white matter bundles embedded in the SLF); (b) Evidence from lesion studies in patients with visuospatial attentional deficits and hemineglect; (c) Data‐driven approaches based on evidence obtained for the previously mentioned hypothesis‐driven coalitions of white matter bundles. Note that for the selected bundles of our study, only voxels encompassing white matter areas (avoiding at all times their cortical projection sites or transition zones) were considered for further analyses. A final ROI representing the rest of the brain (referred to as RoB) was added, to account for the total size/volume of the lesion affecting other white matter regions than those primarily included in the analyses and to avoid missing potential significant contributions from ROIs not considered in the selected set described above. Note that high contributions of the RoB ROI would strongly indicate that we have neglected important white matter tracts when selecting the players of our coalition. Prior to conducting MSA analyses described in the sections below, we estimated the percentage of injury of each white matter bundle of interest and for the RoB. Note that in the present study, we did not consider the total size of the lesion, as is usually done for structural MRI based voxel‐based lesion‐symptom mapping (VLSM) approaches. Alternatively, the RoB was used to estimate the influence of lesion size on bundles not considered among the coalition of players included in our analyses.

### 2.4 Lesion mask delineation and estimation of the extent of injuries

High‐resolution 3D T1‐weighted anatomical SPGR (spoiled gradient recalled) image datasets were obtained for each patient on a 3 T MRI scanner (General Electrics) at the Babinski clinical facility of the Pitié‐Salpêtrière Hospital, with a standard head signal reception coil and the following parameters: RT (repetition time) = 7,164 ms; TE (echo time) = 3,124 ms; inversion time = 380 ms; flip angle = 15°; acquisition matrix = (0, 288, 256, 0); voxel resolution = 0.5 × 0.5 × 1.2 mm^3^; slice thickness = 1.2 mm; spaces between slices = 1.2 mm.

Using the MRIcro software (Rorden & Brett, 2000) and a graphic tablet (A6 WACOM Intuos), lesion masks were manually delineated and segmented by expert personnel (trained in clinical neuroimaging and neuroanatomy) on each relevant section of the raw 3D T1 MRI volume for each patient. 3D T1 images and lesion masks were reoriented in order to match the standard MNI 152 atlas. We then used the N4ITK tool implemented in ANTS (Tustison et al., 2010) and completed intensity bias correction, ignoring the segmented lesion. A binary mask of the brain was computed by registering the MNI 152 atlas to the T1 native space. Registration was assessed outside the stroke‐damaged area, with a rigid, affine and symmetric diffeomorphic transformations, by masking the lesion. This set of transformations was applied to the binary mask of the brain in the MNI 152 space to assess the brain mask in T1 native space.

Due to deterioration of the registration performance caused by damaged tissue within the lesion area, and in order to perform an accurate registration of the MNI 152 atlas into high‐resolution T1, we employed an enantiomorphic registration approach (Nachev, Coulthard, Jager, Kennard, & Husain, 2008). This procedure fills the lesion with the homologous region in the contralateral healthy hemisphere before undergoing coregistration. The high‐resolution lesion‐filled T1 MRI image was then used as a reference to achieve rigid, affine and symmetric diffeomorphic registrations of the MNI 152 atlas (1x1x1 mm3) in the T1 native space.

In order to conduct the MSA analyses described in the sections below, we estimated the percentage of injury of each selected right hemisphere white matter bundle. To this purpose, the previous linear and nonlinear transformations were applied to a probabilistic atlas of white matter fibers in the MNI 152 space (Foulon et al., 2018). This procedure allowed us to compute the probability of an individual voxel to belong to a specific white matter bundle. In order to assess quantitatively the impact of the lesion on the main fiber bundles, we defined a disconnection metric. This was formalized as *d*(*lesion*, *bundle*) and measured the proportion of damaged tissue within a specific bundle using binary segmentation of the white matter fibers bundle (see Equation 1), where # denotes the number of voxels:

(1) $$d\left(\text{lesion},\text{bundle}\right)=\frac{\#\left(\text{lesion}\cap \text{bundle}\right)}{\#\text{bundle}}$$

We calculated the absolute number of voxels for each right hemisphere white matter bundle that was also part of the lesion mask, divided by the total absolute number of voxels present on each white matter bundle, multiplied by 100. The percentage of lesioned voxels for the RoB was computed as the sum of the absolute lesion volumes for the regions not considered in the analyses, divided by the sum of the absolute volumes of these regions, taken together.

### 2.5 Multiperturbation Shapley value analysis

In the MSA framework, the system elements represented by the selected white matter bundles, that is, the 11 white matter bundles and the “RoB” selected in our study, can be conceived of as players in a coalition game where a “perturbation configuration” represents a subset of elements, which are “perturbed” concomitantly. The group of white matter bundles that are left intact (or “uninjured”) represents the coalition of players. For each configuration, we measured the performance of the system on a given set of behavioral tasks.

The analysis aims to assign values representing white matter bundles contribution to (or importance for) task performance. The contribution value of a player, formalized as the Shapley value (Shapley, 1953), represents the difference between the worth of coalitions which contain the element, and the worth of coalitions which do not contain it. When all possible binary perturbation configurations and corresponding performance scores are known, the Shapley value can be computed with the coalition‐based MSA (for further details about the use of this methods in stroke data see also Toba et al., 2017).

In the present study, we applied the so‐called “estimated” MSA which is suitable for large systems consisting of many (up to 100) individual elements (Keinan, Sandbank, Hilgetag, Meilijson, & Ruppin, 2006). This approach is generally used when the number of system elements is too large to enumerate all configurations in a straightforward manner. In this specific MSA approach, the computation alternatively samples orderings and calculates an unbiased estimator of the contribution of each element. In the estimated MSA, a prediction model is trained using a given available set of perturbation configurations and their associated performance scores. Then, this set is used to predict the performances of any perturbation configuration generated by the sampled permutations.

### 2.6 Data preparation for MSA

The dataset used for MSA computations was derived from *line bisection*, *bells cancellation*, and *letter cancellation* outcomes. For each patient, the graded measure of relative white matter lesion size (percentage of disconnection) for each selected bundle and for the RoB was associated with the binarized performance score representing two binary outcomes of each respective test (0 indicating “normal” performance and 1 signaling “pathological” performance). Specific clinical standardized cut‐off levels were used in order to binarize behavioral scores. As mentioned above (Section 2.2), specific clinical standardized cut‐off levels defined by the French Guidelines established by the GEREN (*Groupe d'Étude sur la Rééducation et l'Évaluation de la Négligence unilatérale*) for the tests of the BEN (*Batterie d'Évaluation de la Négligence unilatérale*) evaluation battery were used in order to binarize behavioral scores: scores larger than two in absolute value in the bells and letter cancellation (for ages above 50 years), and scores higher than +6.5 mm or smaller than −7.3 mm in line bisection (Rousseaux et al., 2001). Since binary scores represent the severity of neurologic deficit and MSA requires a score representing behavioral ability, we used the inverse of each score as an indicator of functional performance (1 – *current score*).

The original dataset, composed of ~25 graded stroke lesion configurations (describing relative lesion size) for the 11 areas and the RoB (i.e., a total of 11 + 1 = 12 white matter bundles or players) and their associated performance scores were far from representing the full set of all possible combinations of binary states, as is typically the case for opportunistic samples (*original‐graded dataset*). Indeed, due to the large number of white matter “players” (11 + 1 = 12) and the small number of patients (~25), we had to apply the “estimated” MSA method which predicts binary performance scores for any perturbation configuration generated by 1,000 sampled permutations. A machine‐learning model for binary classification, and a support vector machine, SVM (Matlab, Mathworks), were trained on the available input original‐graded dataset (training dataset) of ~25 stroke clinical cases, each characterized by a unique graded lesion pattern, spanning from 0 to 1, (“0” indicating complete damage of a white matter tract and “1” indicating its complete preservation or sparing from damage), and the corresponding binary scores for each neuropsychological test (training labels).

We first assessed the statistical power of the binary predictor by computing a *classification* (*prediction*) *accuracy* applying a “leave‐one‐out” cross‐validation on the original‐graded dataset for every test, using in turn each single case from the training data as the *validation data* and all the remaining cases as the *training data*. Classification accuracy was computed by counting the number of successful predictions (i.e., the number of times that the real binary score was predicted correctly) in the “leave‐one‐out” cross‐validation. In this procedure, a value of 100% indicates that the SVM correctly predicted the scores for all the ~25 clinical cases. The SVM method used to find the separating hyperplane was the sequential minimal optimization.

The parameter representing the box‐constraint factor for the SVM was set to default value (*c* = 1). A SVM with linear kernel was used for the *line bisection*, whereas a SVM with polynomial kernel was implemented for the *bells cancellation* and a SVM with quadratic kernel for the *letter cancellation*. This choice was made in order to maximize classification accuracy for each individual neuropsychological test after a sensitivity analysis of the SVM's kernel parameter. The result of this process yielded prediction classification accuracies of 91%, 75%, and 68% for *line bisection*, *bells cancellation*, and *letter cancellation*, respectively. These levels proved considerably higher than their respective statistical chance levels (52%, 55%, and 50%) computed as the classification accuracies (i.e., leaving out in turn each single case), but using randomly permutated instead of predicted scores. More specifically, we left out on each turn, each single case from the original dataset and compared the actual value to the corresponding randomly permutated score obtained by shuffling, instead of using the predicted score. We repeated this procedure 1,000 times and averaged the values across the pool of 1,000 samples.

An additional performance measure to evaluate the reliability of the binary prediction process, the *Youden index* (Youden, 1950), was also computed. This estimate is calculated as the *sensitivity* + *the specificity* − 1 and its value ranges from −1 to 1. A value of 1 indicates that there are no false positives or false negatives in the prediction. The Youden index for our battery of clinical scores was 0.8, 0.6, and 0.4 for *line bisection*, *bells cancellation*, and *letter cancellation*, respectively. These values were considerably higher than the respective Youden index computed with randomly permuted scores (~0 for the three neuropsychological tests).

**Figure 1:**
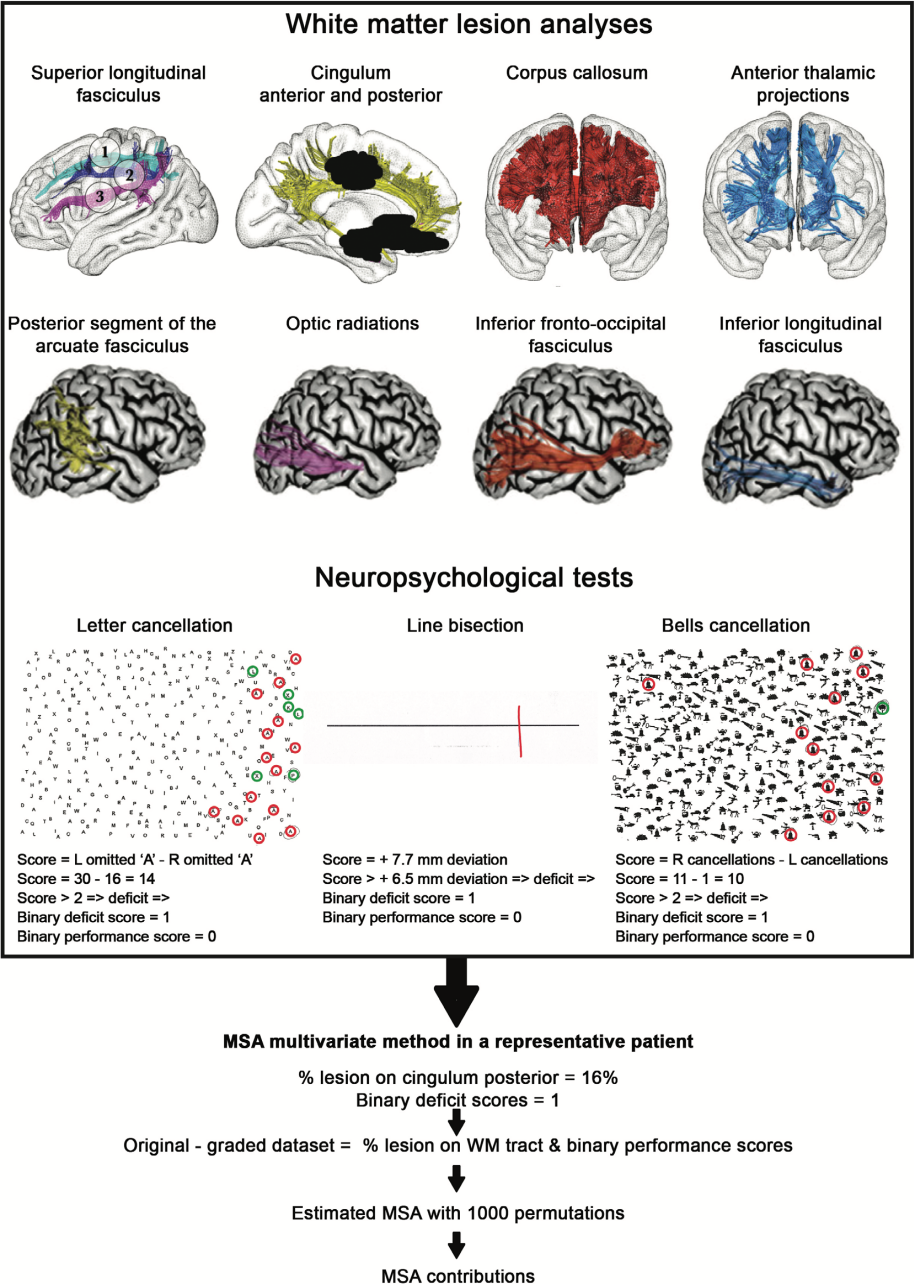
Schematic representation of the methodological pipeline of our MSA study in a representative patient. The lesion analysis panel shows the 11 white matter bundles considered in this study. First row from left to right: the three branches (from dorsal to ventral) of the superior longitudinal fasciculus (SLF I, SLF II, SLF III), the anterior and posterior portions of the cingulum (CA and CP), the corpus callosum (CC) and the anterior thalamic projections (ATP); second row from left to right: the posterior segment of the arcuate fasciculus (APS), the optic radiations (OR), the inferior fronto‐occipital fasciculus (IFOF) and the inferior longitudinal fasciculus (ILF). Notice in this example panel the presence of a lesion mask (in black) encompassing parts of the cingulum bundle. The percentage of damage was estimated for each of these tracts. The “Neuropsychological evaluation tests” section shows patient's behavioral performance in the *bells cancellation*, *line bisection*, and *letter cancellation* tests (red circles represent patients' correct performance, while green circles represent distractors mistaken as targets) and the binarization of the score (deficit = 0, normal performance = 1). The MSA approach included the generation of the original‐graded dataset, the application of the estimated MSA with 1,000 sampled permutations that we performed 1,000 times with bootstrap samples and the computation of each white matter tract contributions. MSA, multiperturbation Shapley value analysis; WM, white matter; L, left; R, right *Source:* Illustrations are adapted from Rojkova et al. (2016) and Urbanski et al. (2011)

We subsequently obtained the contributions of each player in the coalition via 1,000 samples of bootstrapping the estimated MSA approach with 1,000 sampled permutations for the *line bisection*, *bells cancellation*, and *letter cancellation* tasks (the entire methodological process is summarized in Figure 1). The bootstrapping was used to ensure the robustness of the obtained contributions and to estimate the associated *SE*. Essentially, from the available dataset, we chose 1,000 random samples with replacement, with the size of the original dataset. We then performed the estimated MSA on each of these 1,000 samples. Finally, the contributions and *SE*s were averaged across the pool of 1,000 samples. Note that the method used in Toba et al. (2017) included the computation of interactions between gray matter areas. These interactions are not structurally and functionally plausible between white matter bundles, hence we did not consider such interactions in the present analysis.

## 3 RESULTS

We first computed the relative lesion size (graded from 0% to 100% of lesion) for the 11 white matter bundles of interest of the right hemisphere selected for the study: the SLF with its three branches (SLF I, SLF II, SLF III), the IFOF, the ILF, the CC, the ATP, the OR, the APS, the CA, the CP, and the RoB.

**Figure 2:**
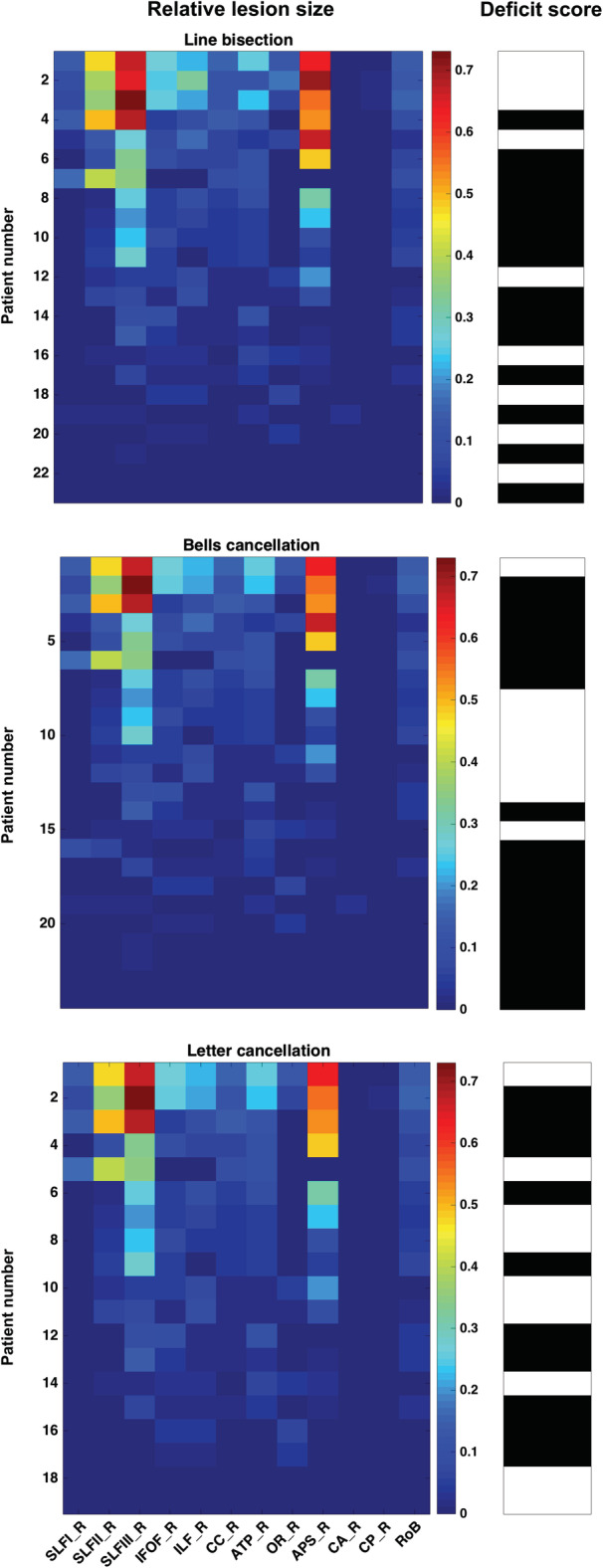
Patterns of relative lesion sizes of white matter regions and associated binary neuropsychological scores across patients. Panels represent the relative lesion size (in % of white matter damaged voxels with respect to the total number of voxels associated to each tract) for each of the 11 white matter bundles considered in the analyses, and an additional white matter ROI representing the rest of the brain (RoB), that is, a compound of any white matter area not included in the 11 bundles of our study. The three panels correspond to patients evaluated with each of the three neuropsychological tests performed by the patients: *line bisection* (*n* = 23 patients), *bells cancellation* (*n* = 24 patients), and *letter cancellation* (*n* = 19 patients). Relative lesion patterns are associated with binary performance scores. For each of the three neuropsychological tests, individual cases (patients) are shown sorted in descending order, from the largest to the smallest lesion size. The color‐coded scale displays the relative lesion size (from the lowest (0%) to the highest (88%) percentage of damaged voxels, color‐coded scale from blue to red hues). Binarized scores representing task performance values for the three neuropsychological tests are represented in black (0 = normal performance) versus white (1 = pathological performance)

These values were associated (Figure 2) with binarized scores (0 for “normal” or “absence” of deficit displayed in black color or 1 signaling “pathological level of impairment” represented in white) for the three neuropsychological tests (*line bisection*, *bells cancellation*, and *letter cancellation*). These calculations showed that for our selected set of 11 bundles, deficit scores were associated with different lesion sizes. More specifically, patients with large lesions and patients with small lesions affecting a given bundle presented pathological scores according to the *cut‐off* levels used to classify test scores. For all the tests, pathological score values were widely spread across patients, who showed or not lesions of different levels of severity encompassing the white matter bundles considered in our analyses.

**Figure 3:**
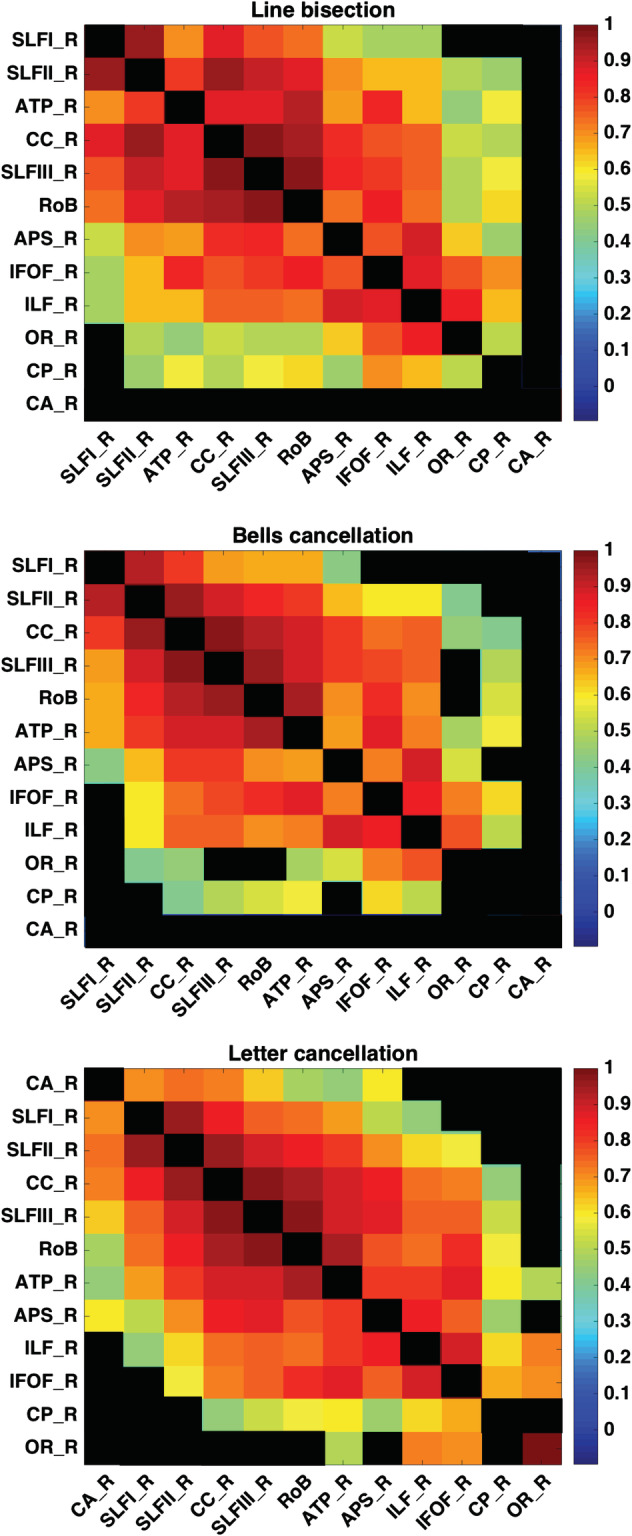
Correlations between lesion patterns across white matter bundles for patient samples corresponding to the three neuropsychological tests considered in the study. Correlations between regional damage levels patterns across the 11 white matter bundles, calculated separately for each subcohort of patients evaluated in each of the three neuropsychological tests (*line bisection*, *n* = 23 patients, *bells cancellation*, *n* = 24 patients, and *letter cancellation*, *n* = 19 patients). The strength of the correlation across pairs of ROIs is displayed. Statistically significant correlations (*p* < .05) are represented in graded colors from lowest (blue) to highest (red) correlation coefficient, whereas nonsignificant correlations are blacked out.

We then computed *pairwise* Pearson correlation coefficients using Matlab (Mathworks, MA; Figure 3) for the relative regional lesion patterns (i.e., correlations between all pairs of relative lesion sizes, specifically sorted for each task to present the highest values close to the diagonal) for the selected coalitions of white matter bundles across patients (*n* = 23, *n* = 24, and *n* = 19), which were evaluated separately for each of the three neuropsychological tests (*line bisection*, *bells cancellation*, and *letter cancellation*). These analyses assessed the level of covariance in lesion patterns across white matter bundles, caused by their dependence on a common source of blood supply (i.e., colocalization within the same vascular territory). Results suggest largely independent lesion patterns (i.e., lesion size correlation coefficients <0.5) (Figure 3).

**Figure 4:**
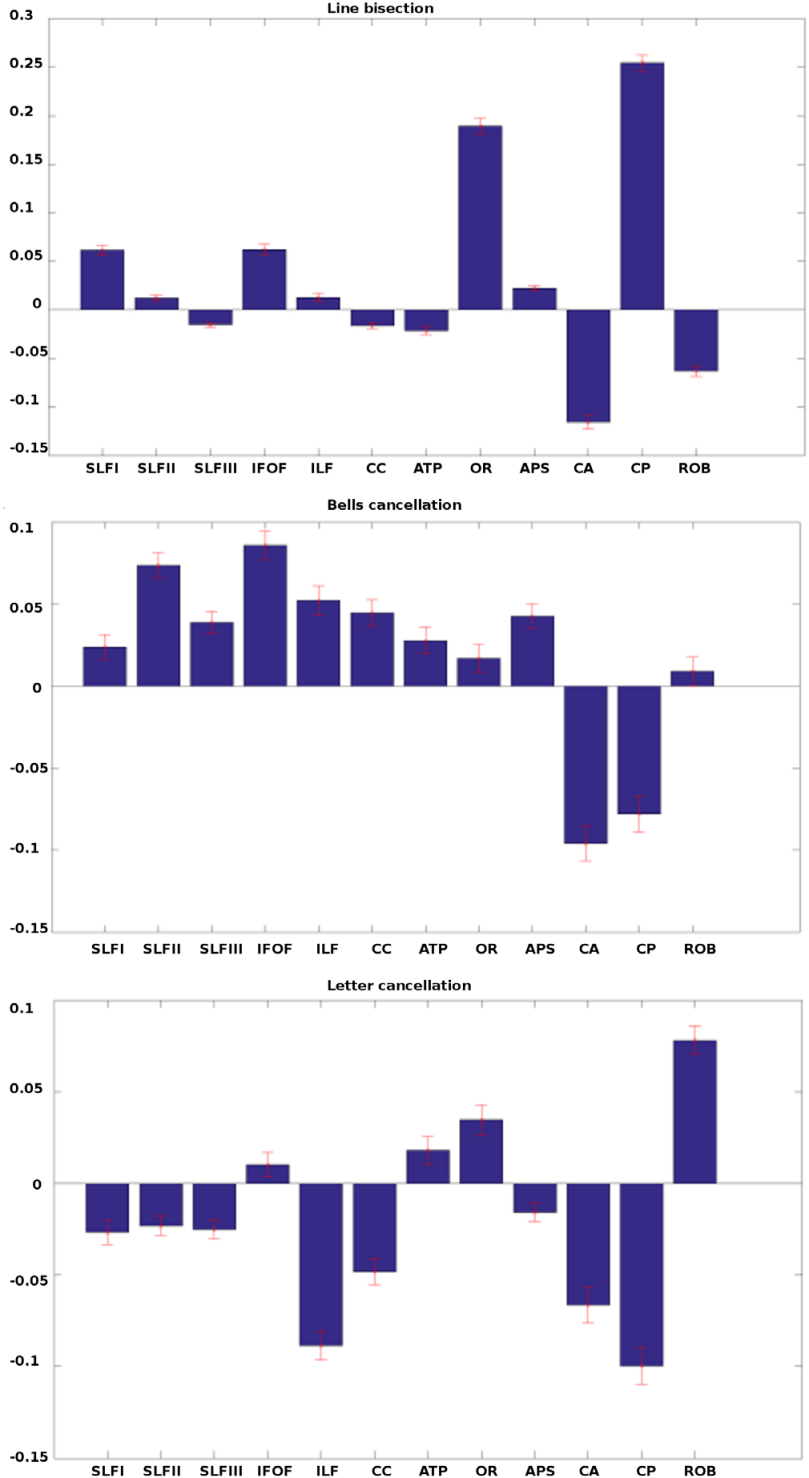
MSA contributions to visuospatial attentional orienting processes of white matter tracts for each of the three neuropsychological tasks. Normalized mean MSA contribution values (± *SD*) for *line bisection*, *bells cancellation*, and *letter cancellation*, respectively (top, middle, and bottom). Values are computed using the original‐graded dataset based on machine‐learning predictions of performance scores, corresponding to configurations generated with 1,000 sampled permutations, and a bootstrap procedure with 1,000 samples to improve the robustness of the results (contributions and estimation of error). Positive values indicate positive contributions (i.e., damage of the white matter bundle leads to decreases of performance in the evaluated task). Negative values signal negative contributions (i.e., damage of the white matter bundle results in increased performance)

In order to characterize the role of each bundle in the three tests, we computed the mean estimated MSA contribution values across 1,000 bootstrap samples (Figure 4) by predicting the behavioral scores for any perturbation configuration generated by 1,000 sampled permutations. *SD* bars were derived with the “estimated MSA” approach averaged across the 1,000 bootstrap samples (see Section 2.6).

MSA values were all significantly different from 0. Positive contributions indicate that a white matter bundle facilitates performance in a given neuropsychological test; thus, if this is injured, performance would decrease. In contrast, negative contributions indicate that a given bundle hinders task performance; hence, damage of such tract may actually improve neuropsychological performance scores.

Our analyses showed that the contributions of right hemisphere white matter bundles differed for the three neuropsychological tests. The SLF, the OR, the IFOF and the CP tracts provided the strongest positive contributions. We could characterize task‐invariant (i.e., positive or negative for all tasks together) contributors, that is, white matter bundles contributing significantly to all the tasks with the same type of contribution (positive or negative), and task‐dependent (i.e., either positive or negative for each individual task) contributors, that is, a white matter bundle contributing significantly (positively or negatively) to one single task.

For instance, the OR and the IFOF were task‐invariant positive contributors. Focusing on positive contributions to each individual neuropsychological test, the SLF I, IFOF, OR, and CP provided the largest positive influences to *line bisection*; the remaining contributions were numerically very weak. For the *bells cancellation*, the IFOF, the SLF II, the ILF, the CC, the APS, the SLF III, and the ATP showed (from highest to lowest) the greatest positive contribution to performance. For the *letter cancellation*, the strongest positive contributors were the ATP, the OR, and the RoB ROI.

The only task‐invariant negative contributor was the CA. The other negative contributors were task‐dependent. For *line bisection*, any other negative contributions proved very weak. For the *bells cancellation*, the CP provided the other negative contribution. Finally, for the *letter cancellation* task, the CP, the ILF, the CC, and the APS exerted other negative contributions. All these results are synthetized in Table 2 and a full set of neurological examples can be found in Table 3.

**Table 2:** Synthesis of the contributions obtained with the game theory‐based MSA approach in white matter tracts (present study) and gray matter regions (Toba et al., 2017) for the three neuropsychological tests used to explore attentional orienting

Contributions	Studies conducted in these same patient cohort	Neuropsychological tests		
		Line bisection	Bells cancellation	Letter cancellation
Positive contributions	White matter (current study)	SLF I, SLF II, IFOF, ILF, OR, APS, CP	SLF I, SLF II, SLF III, IFOF, ILF, CC, ATP, OR, APS	IFOF, ATP, OR
	Gray matter (Toba et al., 2017)	BA6/FEF, BA7/IPS, BA19/IOG, BA 39/TPJ, BA40/TPJ	BA7/IPS, BA39/TPJ, BA45/IFG	BA7/IPS, BA39/TPJ, BA40/TPJ, BA45/IFG
Negative contributions	White matter (current study)	SLF III, CC, ATP, CA	CA, CP	SLF I, SLF II, SLF III, ILF, CC, APS, CA, CP
	Gray matter (Toba et al., 2017)	BA44/IFG, BA45/IFG	BA6/FEF, BA19/IOG, BA44/IFG	BA6/FEF, BA19/IOG, BA44/IFG
No contribution	White matter (current study)	–	–	–
	Gray matter (Toba et al., 2017)	–	BA40/TPJ	–

*Note:* APS, posterior segment of the arcuate fasciculus; ATP, anterior thalamic projections; BA, Brodmann area; CA and CP, respectively, anterior and posterior portions of the cingulum; CC, corpus callosum; FEF, frontal eye field; IFG, inferior frontal gyrus; IFOF, inferior fronto‐occipital fasciculus; ILF, inferior longitudinal fasciculus; IOG, inferior occipital gyrus; IPS, intraparietal sulcus; OR, optic radiations; SLF, superior longitudinal fasciculus with the three branches from dorsal to ventral (SLF I, SLF II, SLF III); TPJ, temporo‐parietal junction.

**Table 3:** Parallels between the results from the present study identifying white matter contributions to visuospatial attention tasks used to assess hemineglect and similar outcomes reported in Toba et al. (2017), concerning gray matter regions contributions linked by such white matter tracts to the same tests

White matter tracts	Gray matter regions linked by white matter tracts	Type of contribution in tests used in the present study	Functional contributions of white matter tracts reported in other studies	Findings from Toba et al. (2017)	
				Gray matter regional contributions	Gray matter regional interactions
First branch superior longitudinal fasciculus (SLF I)	Precuneus and Superior parietal lobule (BA5, BA7) with Superior frontal and anterior cingulate gyri (BA8, BA9, BA32) (Petrides & Pandya, 1984; Thiebaut de Schotten et al., 2011)	– Positive contribution to *line bisection* and *bells cancellation* – Negative contribution to *letter cancellation*	Attentional orienting (Chica et al., 2011 TMS^a^; Doricchi & Tomaiuolo, 2003 ls; He et al., 2007 ls; Shinoura et al., 2009 ls; Thiebaut de Schotten et al., 2014 ls; Thiebaut de Schotten et al., 2005 as; Urbanski & Bartolomeo, 2008 ls; Roux et al., 2011 as) Modulation of conscious visual performance (Chanes et al., 2012 TMS; Chanes et al., 2013 TMS; Quentin et al., 2015 TMS; Quentin et al., 2016 TMS) Movement planning, oculomotor coordination and visual reaching (Anderson et al., 2012 MRI; Leiguarda & Marsden, 2000) Visual near‐threshold detection performance and voluntary top down orienting of spatial attention (Quentin et al., 2015 TMS; Quentin et al., 2016 TMS) Language articulation (Rolland, Herbet, & Duffau, 2018 as)	**BA7/IPS** – Positive contributor to the three tasks: *line bisection*, *letter cancellation*, and *bells cancellation* **BA6/FEF** b – Positive contributor to *line bisection* – Negative contributor to *bells cancellation* and *letter cancellation*	**Synergistic** BA7/IPS–BA6/FEF^b^ (*line bisection*)
Second branch superior longitudinal fasciculus (SLF II)	Anterior intermediate parietal sulcus and Angular gyrus (BA39, BA40) with posterior regions of superior frontal gyrus and middle frontal gyrus (BA8, BA9) (Petrides & Pandya, 1984; Thiebaut de Schotten et al., 2011)	– Positive contribution to *line bisection* and *bells cancellation* – Negative contribution to *letter cancellation*		**BA6/FEF** b – Positive contributor to *line bisection*, negative contributor to *bells cancellation*, and *letter cancellation* **BA39/TPJ** – Positive contributor to the three tasks*: line bisection*, *letter cancellation*, and *bells cancellation* **BA40/TPJ** – Positive contributor to *line bisection* and *letter cancellation*	**Synergistic** BA39/TPJ–BA40/TPJ (*line bisection*, *bells*, and *letter cancellation*) BA39/TPJ–BA6/FEF^b^ (*line bisection*) BA40/TPJ–BA6/FEF^b^ (*line bisection*) BA6/FEF^b^–BA7/IPS (*line bisection*) BA7/IPS–BA39/TPJ (*line bisection*, *bells cancellation*) BA7/IPS–BA40/TPJ (*line bisection*) **Redundant** BA39/TPJ–BA6/FEF (*letter cancellation*)
Third branch superior longitudinal fasciculus (SLF III)	Temporo‐parietal junction (BA40) with inferior frontal gyrus (BA44, BA45, BA47) (Petrides & Pandya, 1984; Thiebaut de Schotten et al., 2011)	– Positive contribution to *bells cancellation* – Negative contribution to *letter cancellation* and *line bisection*		**BA40/TPJ** – Positive contributor to *line bisection* and *letter cancellation* **BA44/IFG** – Negative contributor to all three tasks*: line bisection*, *letter cancellation*, and *bells cancellation* **BA45/IFG** – Negative contributor to *line bisection*, positive contributor to *bells*, *cancellation*, and *letter cancellation*	**Synergistic** BA45/IFG–BA40/TPJ (*bells cancellation*) BA39/TPJ–BA 45/IFG (*bells* and *letter cancellation*) BA39/TPJ–BA40/TPJ (*line bisection*, *bells*, and *letter cancellation*)
Cingulum anterior (CA) and cingulum posterior (CP)	**Longest Fibers** Amygdala, uncus (BA35), Parahippocampal gyrus (BA36, BA30) with Subgenual areas of the orbitofrontal lobe (BA25, BA11) **Shorter fibers** Connect with adjacent areas of the cingulate cortex (BA23, BA24), superior medial frontal gyrus (BA32, BA6, BA8, BA9), paracentral lobule (BA4), precuneus (BA7), cuneus (BA19), lingual (BA18, BA19), and fusiform gyri (BA19, BA37) (Catani, 2016)	– Positive contribution of CP to *line bisection* – Negative contribution of CP to *bells cancellation* and *letter cancellation* – Negative contribution of CA to all three tests: *line bisection*, *letter cancellation*, and *bells cancellation*	**Cingulum anterior (CA)** Attention, memory and emotions (Rudrauff et al., 2008 ls; Catani, 2016) Working memory, sensory‐driven attention, theory of mind, prospective and autobiographic memory (Amodio & Frith, 2006; Broyd et al., 2009; Catani, 2016; Raichle & Snyder, 2007) Ventral portion of the cingulum involved in spatial orienting (Aggleton, 2008 ls; Catani, 2016; Vann, Aggleton, & Maguire, 2009) Motivational deficit in orienting of attention (“motivational” neglect) (Lecce et al., 2015 ls) Executive functions (Burks et al., 2017 as) **Cingulum posterior (CP)** Spatial attention and motor neglect (Garbarini, Piedimonte, Dotta, Pia, & Berti, 2013 ls; Migliaccio et al., 2014 ls; Umarova et al., 2014 ls) Sustained attention (Bonnelle et al., 2011 ls)	**BA6/FEF** b – Positive contributor to *line bisection* – Negative contributor to *bells cancellation* and *letter cancellation* **BA7/IPS** – Positive contributor to all three tasks: *line bisection*, *letter cancellation*, and *bells cancellation* **BA19/IOG** – Positive contributor to *line bisection*, negative contributor to *bells cancellation* and *letter cancellation*	**Synergistic** BA7/IPS–BA19/IOG (*line bisection*, *bells cancellation*) BA6/FEF^b^–BA7/IPS (*bells cancellation*) BA6/FEF^b^–BA19/IOG (*line bisection*) **Redundant** BA6/FEF^b^–BA19/IOG (*bells* and *letter cancellation*)
Corpus callosum (CC)	The anterior portion (*genu*) connects the prefrontal and orbitofrontal regions; the central portion (*body*) connects precentral frontal regions and parietal lobes; the posterior portion (*splenium*) connects the occipital lobes and the temporal lobes (tapetum) (Catani & Thiebaut de Schotten, 2008)	– Positive contribution to *bells cancellation* – Negative contribution to *line bisection* and *letter cancellation*	Transferring of inputs from one hemisphere to the other; motor, perceptual, cognitive functions (Glickstein & Berlucchi, 2008; Doron & Gazzaniga, 2008; Balsamo, Trojano, Giamundo, & Grossi, 2008 ls) Attentional orienting (Bozzali et al., 2012 ls; Lunven et al., 2015 ls; Vaessen et al., 2016 ls)	Not specifically analyzed	Not specifically analyzed
Anterior thalamic projections (ATP)	Cross the internal capsule, and enter the *corona radiata* terminating in the cortex of the ipsilateral hemisphere; radiate anteriorly to the frontal cortex (anterior thalamic peduncle), superiorly to the precentral frontal regions and parietal cortex (superior thalamic peduncle), posteriorly to the occipito‐temporal cortex (posterior thalamic peduncle), and inferoanteriorly to the temporal cortex and amygdala (inferior thalamic peduncle) (Catani et al., 2016)	– Positive contribution to *bells cancellation* and *letter cancellation* – Negative contribution to *line bisection*	Attentional orienting (Cambier, Elghozi, & Strube, 1980 ls; Mesulam, 1999; Rafal & Posner, 1987 ls; Schott, Laurent, Mauguiere, & Chazot, 1981 ls; Watson & Heilman, 1979 ls; Thiebaut de Schotten et al., 2014 ls)	Not specifically analyzed	Not specifically analyzed
Optic radiations (OR)	Lateral geniculate nucleus to primary visual cortex (BA17) (Urbanski et al., 2011)	– Positive contribution to all three tests: *line bisection*, *bells cancellation*, and *letter cancellation*	Visual perception and orienting of attention (Doricchi & Angelelli, 1999 ls; Urbanski et al., 2011 ls; Rolland et al., 2018 as)	**BA19/IOG** – Positive contributor to *line bisection*, negative contributor to *bells cancellation*, and *letter cancellation*	Not specifically analyzed
Posterior segment of the arcuate fasciculus (APS)	Wernicke territory (posterior part of the superior temporal gyrus and middle temporal gyrus) to Inferior parietal lobule (BA39 and BA40) (Urbanski et al., 2011)	– Positive contribution to *line bisection* and *bells cancellation* – Negative contribution to *letter cancellation*	Attentional orienting (Urbanski et al., 2008 ls) Working memory and language (Geldmacher, Quigg, & Elias, 2007 ls; Husain et al., 2001 ls; Tanabe et al., 1987 ls; Wojciulik, Husain, Clarke, & Driver, 2001 ls; Wojciulik, Rorden, Clarke, Husain, & Driver, 2004 ls; Yamada et al., 2007 ls) Time‐locked integration of spatial and perceptual information necessary for attentional selection and conscious processing of visual objects (Robertson, 2003)	**BA39/TPJ** – Positive contributor to all three tasks: *line bisection*, *letter cancellation*, and *bells cancellation* **BA40/TPJ** – Positive contributor to *line bisection* and *letter cancellation*	**Synergistic** BA39/TPJ–BA40/TPJ (*line bisection*, *bells*, and *letter cancellation*)
Inferior fronto‐ occipital fasciculus (IFOF)	Inferior and medial surface of the occipital lobe (BA19 and BA18) to ventrolateral frontal cortex (BA11), frontal pole (BA10) and superior frontal gyrus (rostral portion of BA9) (Catani et al., 2016). A part of this bundle could also be associated to (involve fibers of) the extreme capsule fasciculus (ECF) with rostral projections in the BA44 and BA45 (see Petrides, Tomaiuolo, Yeterian, & Pandya, 2012; Umarova et al., 2010)	– Positive contribution to all three tests: *line bisection*, *bells cancellation*, and *letter cancellation*	Reading (Epelbaum et al., 2008 ls) Visual processing (Fox, Iaria, & Barton, 2008 ls; Rudrauff et al., 2008 ls) Visuospatial attention (Herbet, Yordanova, & Duffau, 2017 as; Toba, Migliaccio, et al., 2018 ls; Urbanski et al., 2008 ls; Vaessen et al., 2016 ls; Roux et al., 2011 as; Doricchi et al., 2008)	**BA19/IOG** – Positive contributor to *line bisection*, negative contributor to *bells cancellation*, and *letter cancellation*	Not specifically analyzed
Inferior longitudinal fasciculus (ILF)	Short/long fibers connecting visual areas (extrastriate areas, posterior lingual and fusiform gyri, medial regions of the cuneus) with middle and inferior temporal gyri and temporal pole, parahippocampal gyrus, amygdala, and hippocampus (Catani & Thiebaut de Schotten, 2008; Catani et al., 2016)	– Positive contribution to *line bisection* and *bells cancellation* – Negative contribution to *letter cancellation*	Face recognition (Fox et al., 2008 ls; Corrivetti, Herbet, Moritz‐Gasser, & Duffau, 2017 as) Visual perception (Ffytche, 2008 ls) Reading (Epelbaum et al., 2008 ls; Zemmoura, Herbet, Moritz‐Gasser, & Duffau, 2015 as) Lexical retrieval (Herbet, Moritz‐Gasser, Lemaitre, Almairac, & Duffau, 2018 as) Visual memory (Ross, 2008)	**BA19/IOG** – Positive contributor to *line bisection*, negative contributor to *bells cancellation*, and *letter cancellation*	Not specifically analyzed

Abbreviations: as, awake surgery study; BA, Brodmann area; FEF, frontal eye field; IFG, inferior frontal gyrus; IOG, inferior occipital gyrus; IPS, intraparietal sulcus; ls, lesion study; TMS, transcranial magnetic stimulation; TPJ, temporo‐parietal junction.

a Transcranial magnetic stimulation (TMS) studies cannot probe white matter tracts but only gray matter cortical areas associated to these tracts. However, interindividual performance differences induced by the TMS can be correlated to white matter tracts features even if those do not necessarily project to stimulated cortical regions. Hence, TMS can only provide correlational evidence on potential white matter tract contributions.

b The coordinates of the right frontal eye field (FEF) were associated with BA6 in the study of Toba et al. (2017). This association has been validated in causal brain stimulation studies (Chanes et al., 2012, 2013; Quentin et al., 2015, 2016).

Interestingly, several white matter bundles showed positive contributions to some neuropsychological tests and negative to some others. For instance, the CP bundle made a strong negative contribution to the *bells cancellation* and *letter cancellation* tests, and also positive contribution to *line bisection*. Similarly, the SLF III showed a positive contribution to the *bells cancellation* test, but negative ones for both *line bisection* and *letter cancellation* tests. The SLF II showed a positive contribution to the *bells cancellation* and *line bisection* tests, and a negative contribution to the *letter cancellation*.

In sum, the outcomes of the MSA analyses for white matter tracts allowed us to identify bundles with task‐invariant positive contributions to visuospatial orienting of attention. Also, they allowed us to identify white matter bundles providing task‐invariant negative contributions (see the CA), and others having alternating task‐dependent contributions (see CP, SLF I, SLF II, and SLF III).

In terms of functional localization, the *bells cancellation* and *line bisection* shared several similar patterns of positive/negative contributions (mainly, positive for the IFOF, SLF I, OR and APS and negative for the CA), whereas *line bisection* and *letter cancellation* shared other similar patterns of positive/negative contributions (positive for the OR and the IFOF, and negative for the SLF III, CC, and the CA). *Letter cancellation* and *bells cancellation* shared positive contributions from the ATP, the OR and the IFOF, and negative contributions by the CP and the CA.

One may also note that the RoB, representing the ensemble of white matter bundles not considered individually in the coalition, showed negative contribution to *line bisection* but positive contributions to the *letter* and *bells cancellations* tasks.

## 4 DISCUSSION

In the present study, we applied the multivariate game‐theoretical MSA approach to the characterization of causal contributions of right hemisphere white matter tracts to visuospatial attentional orienting explored with three neuropsychological paper‐and‐pencil tasks. We observed that the OR and the IFOF made task‐invariant positive contributions, whereas the CA contributed negatively to the three visuospatial neuropsychological tests. The other white matter bundles showed task‐dependent contributions. The main positive contributors to *line bisection* were the first branch of the SLF (SLF I), the IFOF, the OR and the CP. For the *bells cancellation* task, the SLF I, SLF II, SLF III, IFOF, the ILF, the CC, the ATP, and the APS displayed the highest positive contribution to performance, whereas for the *letter cancellation* task, the main positive contributors were the ATP, the OR, and the IFOF bundles.

Several structures showed positive contributions to one neuropsychological test and negative to the other two, demonstrating that the same white matter tract could simultaneously facilitate or hinder different aspects of visuospatial attentional performance. The most important dissociations concerned white matter structures such as the CP (showing negative contributions to *bells cancellation* and *letter cancellation* and positive contribution to *line bisection*), the SLF II and the SLF III (both having positive contributions to the *bells cancellation* and negative contributions to *letter cancellation* test).

### 4.1 Common positive or negative contributors to visuospatial attentional tasks

The main task‐invariant positive contributor of this study was the OR, a tract linking the lateral geniculate nucleus to the primary visual cortex. This tract connects two regions on which damage is likely to cause visual field defects such as hemianopia, but not hemineglect. It is well‐known that in right hemisphere stroke lesions leading to hemineglect, hemianopia often coexists and interacts with visuospatial attentional and awareness disorders, worsening patients' performance especially in the acute phase (Cassidy, Bruce, Lewis, & Gray, 1999; see also Weintraub & Mesulam, 1988 who questioned the statistical robustness of such findings). However, hemianopia is very unlikely to produce per se a full hemineglect‐like syndrome in the absence of attentional orienting dysfunctions. The interaction between neglect and hemianopia has been previously identified as important for *line bisection* task (see Doricchi & Angelelli, 1999), whereby patients with both, neglect and hemianopia, showed a larger ipsilesional shift when bisecting horizontal lines compared to patients with neglect but not in those with hemianopia. However, these two groups of patients displayed comparable levels of neglect severity on multiple‐item cancellation tasks. Further studies showed that performance of neglect patients on cancellation tasks using moving targets was influenced by the integrity of the OR (Hopfner et al., 2015). Other authors proposed that hemianopia did not exacerbate neglect, and that poor functional recovery in patients with visual field deficits was caused by an association between visual sensory losses and neglect (Halligan, Marshall, & Wade, 1990). Moreover, a recent study reported a reduction of fractional anisotropy (FA), one of the most common parameters used in DTI studies to estimate the presence of oriented white matter fibers (Le Bihan et al., 2001), in the OR of patients who did not recover spontaneously from attentional orienting deficits (Umarova et al., 2014), suggesting a complex contribution of visual processing systems in attentional orienting functions. Additionally, double dissociations between hemianopia and visuospatial attention deficits (such as hemispatial neglect) are often observed in clinical practice, suggesting that in spite of being a positive contributor to visuospatial tasks, OR disconnections worsen performance only when sharing a coalition of positive contributors in which other bundles and/or gray matter regions are also damaged. In the study at hand, the OR acted as positive contributor to all three tests, suggesting that when this bundle is disconnected, task performance is degraded not only for *line bisection*, but also and quite unexpectedly for *cancellation tasks*. Consequently, further studies should not only focus on pinpointing the detailed role of the OR, but also to further characterize the precise coalitions of positive and negative contributors in the context of which this bundle, when disconnected, worsens the performance of neglect patients. Alternatively, since the present study included patients with hemianopia, we could consider that the so‐ called “disconnection” of a positive white matter contributor such as the OR could have trivial effects in the orienting of attention, thereby highlighting the importance of the other positive contributors such as, for example, the IFOF (see below).

The second task‐invariant positive contributor of this study was the IFOF, a white matter tract connecting the inferior and medial portions of the occipital lobe and of the parietal lobe to the ventral frontal cortex, the frontal pole and the superior frontal gyrus (Catani, Howard, Pajevic, & Jones, 2002; Petrides & Pandya, 1984). Temporal terminal projections have also been characterized (Meynert, 1884). Nonetheless, the identity of the IFOF remains debated. Schmahmann and Pandya (2006) consider that the first horizontal portion of this bundle corresponds to the inferior and medial longitudinal fasciuli, whereas its rostral ascending limb is part of the extreme capsule and projects to the IFG (BA44 and BA45), hence it has been also labeled as the extreme capsule fasciculus (ECF) (Petrides et al., 2012). Umarova et al. (2010) observed that the ventral portion of the IFOF connected the frontal lobe to the temporal and parietal cortices and suggested therefore that its course colocalized with the rostral portion of the extreme/external capsule fiber system. This tract has been characterized as a positive contributor to *line bisection*, linked by several studies with visuospatial attention (Charras, Lupiáñez, Migliaccio, Toba, & Bartolomeo, 2012; Toba, Migliaccio, et al., 2018; Urbanski et al., 2008; Vaessen et al., 2016) and deemed essential for the processing of visual stimuli or convey top‐down influences of the frontal cortex on posterior visual areas. On that basis, perceptual abilities necessary for *line bisection* could explain the main contribution of this white matter bundle to such specific task. We should also emphasize that in our prior MSA lesion study focusing on gray matter regions (Toba et al., 2017), the IOG was identified as a positive contributor to *line bisection*. Given that this region extends across the inferior and medial surface of the occipital lobe (Catani, Jones, & Ffytche, 2005), this result is coherent with the positive contribution of the IFOF here reported.

The IFOF tract showed also a role as positive contributor to the *bells* and *letter cancellation* tests, at difference with strong negative contributions of the IOG (a gray matter structure located on the caudal trajectory of the IFOF), to both *bells* and *letter cancellation* tasks reported in our previous study (Toba et al., 2017). This finding suggests that damage to the caudal projection of the IFOF could paradoxically improve performance in the former task. Summing up, the IFOF exerts positive contribution to attentional orienting components, whereas damage of its caudal portion hinders performance in the cancellation tasks. Given the rostral IFG projections highlighted by Petrides et al. (2012), the contribution of this bundle could also emphasize the role of the ventral attentional network in attentional orienting. Studies to be conducted with intraoperative stimulation during awake brain surgery or the analysis of lesion cases selectively addressing the role of different IFOF portions could shed more light into the functional contributions of this white matter bundle.

The other task‐invariant, but negative, contributor to the tasks analyzed in our study was the anterior portion of the cingulum (CA). Anatomically, the cingulum is a tract containing fibers of different lengths; the longest run from the amygdala, the uncus and the parahippocampal gyrus to subgenual portions of the orbitofrontal cortex; the shortest originating in adjacent areas of the cingulate cortex, project to the superior medial frontal gyrus (BA6 and BA8), the paracentral lobule, and the precuneus, cuneus, lingual and fusiform gyri (Catani et al., 2013; Crosby et al., 1962; Husain & Schott, 2016; Nieuwenhuys et al., 2008). Functionally, different cognitive processes including attentional orienting have been associated with this bundle.

First, given its location, the dorsal cingulum connects areas of the dorsomedial default‐mode network (Raichle et al., 2001; Raichle & Snyder, 2007) that have been associated to working memory and sensory‐driven attentional allocation, but also theory of mind, and prospective and autobiographic memory (Amodio & Frith, 2006; Broyd et al., 2009; Catani, 2016; Husain & Kennard, 1997; Raichle & Snyder, 2007). In addition, the ventral portion of the cingulum (connecting the amygdala and the parahippocampal cortex to retrosplenial cortical regions) is part of a network dedicated to spatial orienting (Aggleton, 2008; Catani, 2016; Mesulam, 1981; Schmahmann et al., 2007; Vann et al., 2009).

Second, it is to note that the anterior cingulate cortex, a projection site of the CA bundle, is important for cognitive control, conflict monitoring during response selection (Botvinick, Braver, Barch, Carter, & Cohen, 2001; Carter et al., 1998; Nieuwenhuis, Yeung, van den Wildenberg, & Ridderinkhof, 2003), control of spatial attention (Morecraft et al., 2012; Morecraft, Geula, & Mesulam, 1993), and reinforcement learning (Rushworth & Behrens, 2008; Silvetti, Alexander, Verguts, & Brown, 2014; Silvetti, Seurinck, & Verguts, 2011). These functional findings are in line with results obtained in the present study suggesting a role of the CA in the orienting of attention and working memory, both necessary to execute the three tasks evaluated in the present study. Additional evidence for the role of the CA in visuospatial attention comes from a recent case report of a male patient displaying a contralesional reward learning deficit (motivational deficit in orienting of attention, or “motivational” neglect). The patient presented with damage in the anterior portion of the cingulum combined with injuries in more lateral cortical and subcortical structures involved in space representation and the orienting of spatial attention (Lecce et al., 2015). On that basis, these authors suggested that this bundle could represent an interface between the limbic system and cortical structures, hence modulating attentional and motor behaviors via motivational inputs.

The identification of the CA bundle as a negative contributor in the present study reappraises the controversy on the role of this tract on attentional orienting. It has been recently suggested that improvement of neglect symptoms after caloric stimulation in the acute phase is associated on the one hand with increased functional connectivity between bilateral vestibular cortical areas, the parahippocampal areas and the dorsal CA, and on the other hand to reduced interhemispheric connectivity between the former and the visual cortex (Conrad, Boegle, Ertl, Brandt, & Dieterich, 2018). These findings highlight the role of a functional network involving the CA and parahippocampal structures in visuospatial attention, probably important for the engagement and modulation of spatial orienting and navigation maps (Best, White, & Minai, 2001; Rowland, Roudi, Moser, & Moser, 2016). In the present study, we only analyzed the contributions of the CA as a structural entity, which came out to be negative. This outcome suggests that improved visuospatial behaviors may be expected when the anterior part of this bundle is disconnected. Further studies should examine additional coalitions involving additional white matter tracts such as the fornix, and to better characterize the role of this association of players in visuospatial behaviors. Finally, the role of this bundle should be ultimately corroborated with evidence from selective lesion cases or interventional stimulation studies.

Positive MSA framework contributions can be easily understood and described. By contrast, negative associations remain more open and can give raise to different interpretations depending on the theoretical model or framework chosen on each case. First, negative contributions to visuospatial attention could be subtended by the Kinsbourne model of interhemispheric rivalry (1977), and linked to inhibitory influences exerted through forebrain commissures. Second, mutually suppressive commissural fibers in the CC or the midbrain are also known to convey inhibitory influences, phenomenon known in feline models as the “Sprague effect” (Sprague, 1966; see Valero‐Cabre, Toba, Hilgetag, & Rushmore, 2020, for a recent review) setting the basis for another connectivity framework in which to interpret negative contributions. Third, in the attention orienting networks model proposed by Corbetta and Shulman (2002), opposing attentional interhemispheric influences were considered important to maintain a dynamic balance between the dorsal and the ventral attention orienting networks via reciprocal inhibition mechanisms, which can be related to the presence of negative contributors such as those revealed in the current MSA study. Intrahemispheric inhibitory interactions (thus the presence of negative contributors) within the attention network are in agreement with this model, since attentional orienting guided by a task (referred to as endogenous, voluntary or task‐ driven processes) often coexists with a conflicting orienting tendency driven in a bottom‐up manner by unexpected environmental stimuli (referred to as exogenous, reflex or stimulus‐ driven processes). Conflict resolution in such case is likely to use inhibition and thus involve, as our MSA findings suggest, the presence of negative contributors.

It should be noted that the MSA approach frequently revealed negative contributions of specific white matter bundles (shown in our case via lesion data) simultaneously to the three neuropsychological paper‐and‐pencil tasks. The negative role of this same kind of associations reported for the CA (extended to the *bells* and *letter cancellation* tests and to *line bisection*) could be subtended by one or several of the above‐mentioned connectivity frameworks. Unfortunately, is out of the scope of our current analyses to tease apart which is more likely or relevant. Moreover, our prior and current study included only neglect patients with right hemisphere stroke lesions (and absence of left hemisphere damage), hence we were unable to use MSA principles to identify any of the above‐mentioned interhemispheric rivalrous interactions. The presently reported inhibitory contributions of white matter bundles to cancellation or line bisection tasks should be further investigated by studies based on different types of behavioral (experimental tasks, not only clinical), neuroimaging (fMRI) or noninvasive (TMS) and invasive (awake neurosurgery stimulation) focal neurostimulation approaches.

### 4.2 Specific positive and negative contributors

#### 4.2.1 Line bisection task

*Line bisection* is one of the most frequently used tests to assess visuospatial attention, involving perceptual (line length estimation), motor (manual bisection), and attentional components (see Toba, Cavanagh, & Bartolomeo, 2011). The principal positive white matter bundles contributors revealed in the present study were the SLF I, the IFOF, the OR, and the CP.

Attentional dorsal and ventral frontoparietal systems, subtended by SLF branches I, II and III, have been repeatedly proven crucial for attentional orienting (Bartolomeo, 2007; Chica et al., 2011; Corbetta & Shulman, 2002; Toba, Rabuffetti, et al., 2018; Toba, Migliaccio, et al., 2018) and the modulation of conscious visual performance (Chanes et al., 2012, 2013; Quentin et al., 2015, 2016). Specifically, in the present study, the SLF I (connecting the superior parietal lobule and the precuneus to the superior frontal gyrus; Thiebaut de Schotten et al., 2011) exerted a positive contribution to *line bisection* performance. This same bundle has also proved relevant in other types of behaviors reminiscent of those necessary in the line bisection task, such as movement planning, oculomotor coordination and visual reaching (Anderson et al., 2012; Husain & Schott, 2016; Johnson & Ferraina, 1996; Leiguarda & Marsden, 2000). Likewise, two separate studies correlating the outcomes of noninvasive stimulation by TMS on the right FEF to modulate spatial attention, found significant correlations between lateralized near‐threshold detection performance and structural features specifically for the right SLF I (Quentin et al., 2015, 2016). Finally, in a prior MSA study conducted in this same population of stroke patients assessing only gray matter regions (Toba et al., 2017), we reported the positive contribution of the FEF, a rostral cortical projection area of the SLF I, to line bisection performance. The current result confirms the role of the SLF I branch in the voluntary top‐down orienting of spatial attention, necessary to succeed in the *line bisection* task.

Our study also revealed a relevant positive contribution of the CP to *line bisection*. Compared to white matter bundles discussed in preceding paragraphs, damage of the CP has been rarely associated with the orienting of spatial attention. Nonetheless, two recent studies (Garbarini et al., 2013; Migliaccio et al., 2014) suggested the involvement of the cingulum in motor neglect, an attentional disorder whereby patients, in spite of normal muscle strength, sound reflex activity and intact sensory capacities, fail to move the limbs contralateral to their brain lesion. It has also been suggested that this bundle could be involved in the redirection of attention, although lesions located at this level rarely result in hemispatial neglect (Mesulam, 2002). Furthermore, Umarova et al. (2014) reported a decreased FA in the CP and the CC in patients that did not recover from attentional orienting deficits. Finally, in patients with traumatic brain injury, a study by Bonnelle et al. (2011) reported a significant correlation between right cingulum damage and impairments of sustained attention. Furthermore, functional connectivity between the posterior cingulate cortex, a projection region of the cingulum bundle, and the default mode network predicted a decline in sustained attention performance. Likewise, it has also been shown that impairments of the sustained component of attention are likely to interact with impairments of the orienting of attention in visuospatial neglect (Robertson, Tegnér, Tham, Lo, & Nimmo‐Smith, 1995). Given that the cingulum links the cingulate cortex with areas of the ventral visual stream and the hippocampal complex, its attentional orienting role could be associated with a more general contribution to the modulation of attentional top‐down control networks, visual processing and memory systems involved in recognition (Rudrauf, Mehta, & Grabowski, 2008).

It should be noted that our results also confirm a role as positive contributors of two cortical regions; the inferior parietal lobule (IPL) and the IOG located caudally along the trajectory of the cingulum and identified as gray matter contributors to similar tasks in our own prior MSA study (Toba et al., 2017). Moreover, in this prior MSA study, the strongest positive interaction for line bisection was found between gray matter regions IOG/BA19 and IPS/BA7, located along the trajectory of the CP. This outcome strongly suggests that these two sites allowed better visuospatial performance when acting jointly than individually (Toba et al., 2017). Complementarily, our current findings support the notion that synergistic interactions between the IOG and the IPS cortical sites are likely mediated by the cingulum and predict a positive role for the CP in *line bisection*, setting the stage for confirmatory evidence to be obtained from lesion single case studies or intraoperative awake surgery stimulation.

The IFOF and OR prove to be also important positive contributors (see above). CA was the only important negative contributor (see above) to *line bisection*. Additional positive contributions (provided by the SLF II and the ILF) and negative contributions (provided by the SLF III, CC, and ATP) emerging from MSA analyses proved very weak, and they will need to be either confirmed or ruled out by further studies conducted on larger samples.

#### 4.2.2 Cancellation tasks

Cancellation tests call upon sustained and selective attention mechanisms necessary to focus and process the targets of any display. Working memory is also essential to maintain a record of the elements that have already been processed, and allocate a maximum of attentional resources to unprocessed targets. Here we observed positive and negative contributions which differed across the two cancellation tasks used in our study, *bells cancellation* and *letter cancellation*.

In the present study, MSA analyses performed with white matter tracts revealed several positive contributors to the *bells cancellation* task: the SLF II, the IFOF, the ILF and the CC. Numerically weak positive contributions to this test were also observed for the SLF III, the ATP, the OR and the APS, hence such potential findings should be taken cautiously and require at this point stronger evidence. The *letter cancellation* task involved only numerically weak positive contributors: the OR, the IFOF (see above) and the ATP. Concerning specific negative contributions to *bells cancellation* and *letter cancellation*, our analyses unearthed a causal hindering role for the CA and the CP for both tasks. Unexpectedly, positive contributors in *bells cancellation* such as the ILF and the CC proved to be negative contributors in *letter cancellation*. Numerically weak negative contributions only for the *letter cancellation* were also observed for all the branches of the SLF and the APS.

The SLF II, connecting the IPS and the angular gyrus (AG) with the posterior regions of the superior and medial frontal gyri, and the SLF III connecting the TPJ with the IFG (Thiebaut de Schotten et al., 2011) has been repeatedly reported as essential for attention (Bartolomeo, 2006, 2007, 2014; Bartolomeo et al., 2007; Corbetta & Shulman, 2011; Doricchi et al., 2008; Doricchi & Tomaiuolo, 2003; He et al., 2007; Shinoura et al., 2009; Thiebaut de Schotten et al., 2014; Thiebaut de Schotten et al., 2005; Urbanski & Bartolomeo, 2008). In our previous MSA study conducted on gray matter regions (Toba et al., 2017), we were able to demonstrate positive contributions of three cortical ROIs such as the IPS, the TPJ, and the IFG located along the trajectory of the SLF II and SLF III branches. By showing strong positive interactions between these gray matter regions, we suggested that these ROIs achieve higher levels of performance when acting jointly than individually (Toba et al., 2017). Here we extend this finding and provide evidence supporting the notion that the synergistic interaction between these cortical sites is subtended by the branches of the SLF.

Second, MSA analyses revealed different contributions of the ILF connecting occipital regions (including the extrastriate visual areas, the posterior lingual and fusiform gyri and medial regions of the cuneus) to the middle and inferior temporal gyri, the temporal pole, the parahippocampal gyrus, the hippocampus and the amygdala. This observation suggests the ability of the ILF to both, facilitate and hinder attentional orienting. The potential role of the ILF on visuospatial attention was first suggested by Bird et al. (2006). However, such findings remained inconclusive and debated as lesion sites in the patients of this study encompassed the trajectory of the IFOF. According to prior reports, the ILF carries information from occipital to frontal regions and is important for visual recognition (objects, faces) and reading (Epelbaum et al., 2008; Ffytche, Blom, & Catani, 2010; Fox et al., 2008). We here note that a gray matter region such as the IOG which is the caudal projection site of the ILF, has previously shown negative contributions to both bells and letter cancellation, adding evidence to the negative contribution of this bundle. These findings challenge once more the role of temporal connections in attention orienting (see Karnath, Ferber, & Himmelbach, 2001; Umarova et al., 2010).

Third, the CC is a white matter interhemispheric commissure that acts as both a positive and negative contributor to attentional orienting in cancellation tasks. Several studies have gathered support for the CC as one of the essential white matter systems for attentional orienting: Bozzali et al. (2012) observed a correlation between hemispatial neglect severity and FA values in the posterior portion of the CC; Umarova, Saur, Glauche, Mader, et al. (2011) concluded an important role for this commissure in the orienting of attention; lastly, Lunven et al. (2015) and more recently Vaessen et al. (2016) associated caudal CC damage to enduring hemispatial neglect. Unfortunately, due to the limited number of parcellations of our white matter atlas (i.e., independent bundles, tracts or commissures), we were unable to assess the contribution of specific CC subregions, a possibility that must be explored to justify mean positive and negative contributions by subportions of this important commissure.

Concerning specific negative contributions to *cancellation tests*, our analyses unearthed a causal hindering role for the CA and the CP. This result remains also to be confirmed by additional evidence from invasive intraoperative explorations in awake neurosurgery patients or lesion cases. Our prior study on gray matter areas also revealed negative contributions to the *bells cancellation* task for the IOG (Toba et al., 2017), a cortical region spanning caudally along the trajectory of the cingulum bundle. This observation strengthens evidence in favor of a hypothetical role for the caudal portion of this bundle as a negative contributor to visuospatial attention. In contrast, other parts of the cingulum, such as for instance projections at the level of areas IPS/BA7 (which showed positive contributions to this task in our prior gray matter MSA study) might contribute differently.

Of note, in the current study, cancellation tests showed different positive and negative contributors. With respect to existing differences between networks involved in performing the different cancellation tests, neuropsychological evidence suggests that the severity of visuospatial attention impairments should not be greater with letters (such as “A”) than with small geometric objects (such as bells) (Gainotti, Perri, & Cappa, 2002). However, a prior study conducted by our team revealed distinct anatomical gray matter contributions patterns to these two tasks. More, specifically, Toba, Migliaccio, et al. (2018) showed positive correlations between the outcomes and common anatomical substrates in VLSM analyses for both the bells and the letter cancellation tests. Nonetheless, along the lines of our current findings, performance in these two tasks correlated with distinct white matter tracts. Although, we can only speculate on such differential contributions, these might reflect distinct operations of the attention network, hence, they could be explained for instance by the orthographic nature of the stimuli employed in the letter cancellation paradigm, which might induce a more systematic left to right visual scanning of the scene, which is not necessarily paralleled in the bells test. Furthermore, ad hoc studies will be needed to further explore and eventually confirm the differential contributions to these two tasks. Lesions selectively impinging on one of these two systems, or awake neurosurgery stimulation able to justify on clinical diagnostic ground the direct stimulation of these white matter regions will prove necessary to causally confirm the robustness of these contributions.

Concerning the SLF contribution, whereas all the branches contributed positively to the *bells cancellation* tasks, they also displayed negative contributions to the *letter cancellation*. As signaled before, this finding emphasizes the fact that the same SLF branches can simultaneously facilitate or hinder different aspects of attentional orienting in space. Compatible with the distribution of these white matter tracts, our prior MSA study reported strong negative contributions to these same tests for cortical gray matter regions such as the right FEF and IFG (Toba et al., 2017), both rostral projection landmarks of the SLF branches. Moreover, since in this same study, the TPJ region displayed positive contributions to similar tasks (Toba et al., 2017), we here put forward the hypothesis that the caudal portion and the rostral portion of this bundle contribute differentially (positively and negatively respectively) to attentional orienting. Ad hoc MSA studies selectively exploring the different portions of the SLF branches, and evidence from per‐operative stimulation in awake neurosurgical patients or noninvasive stimulation approaches in healthy participants need to be conducted in order to further pinpoint functional differences. Note that the contributions of the SLF and the CC, often stronger than those of the OR or IFOF but task‐dependent (i.e., positive or negative depending on the tested task) could subtend different behavioral components of hemispatial neglect. To this regard, it has been shown that this syndrome may be dissociated in different behavioral components, mapped onto different network subsystems within the attentional orienting network (Barbieri & De Renzi, 1989; Binder, Marshall, Lazar, Benjamin, & Mohr, 1992; Charras et al., 2012; Mesulam, 1985; Toba, Rabuffetti, et al., 2018; Vallar, 1998; Wansard et al., 2014), which merge and interact in complex manners (Bartolomeo, 2007; Coulthard, Parton, & Husain, 2007; Gainotti, D'Erme, & Bartolomeo, 1991; Karnath, 1988; Verdon, Schwartz, Lovblad, Hauert, & Vuilleumier, 2010). By employing methodological approaches (i.e., multivariate inference methods) of different nature than those traditionally applied to study brain behavioral association in hemispatial neglect, our study contributes to the characterization of anatomical basis of its clinical components. Furthermore, it identifies modes of interactions between these components, that would need to be studied and confirmed with MRI tractography or noninvasive stimulation technologies.

It should also be noted that the contribution of the RoB ROI, grouping any white matter areas not included in any of our selected set of 11 tracts, proved to contribute positively to *letter cancellation*, hence suggesting that some additional white matter bundles (within the pool of 88 tracts that our hypothesis‐driven and atlas‐based selection strategy provided information on) (Foulon et al., 2018) could exert a facilitatory contribution to this task. Alternative sets of ROIs should be considered in further studies in order to tease out more precisely the optimal selection of white matter tracts (including those not strongly supported by a priori approaches) that might contribute to this and other behavioral clinical evaluation tasks.

### 4.3 Strengths and limitations of the current study

Several methodological aspects need to be considered to accurately interpret the outcomes of the present study. First, we combined *data‐driven* and *hypothesis‐driven* approaches to select and include in each MSA coalition the final set of tracts most likely to play and share a role on each of the three neuropsychological visuospatial tests. For this reason, it was only after observing a relatively important contribution of the RoB value, that we understood the importance of other white matter tracts or tract subregions, notably the OR and the CA and CP, which were not considered in our initial *hypothesis‐driven* selection of white matter players.

Second, using the MSA approach on mean disconnection values for each individual white matter bundle, we were unable to document situations in which similar deficits were induced by lesions on different locations along the trajectory of a bundle, thus to describe very common situations observed in clinical settings, whereby two separate lesions may give rise to the same deficit (see the so‐called “equivalence” brain‐mode reported by Godefroy et al. (1998) and revisited recently in Toba et al., 2019; see also Toba et al., 2020). This situation should be given particular attention in the future. Ruling out whether behavioral deficits result from (a) selective damage to different bundles, (b) damage to different portions along the same bundle, or (c) from the combination of white matter disconnection and gray matter injuries (Doricchi et al., 2008) might prove particularly challenging, but will be essential to refine the accuracy of our MSA inferences.

Third, a limitation of our MSA study concerns the binarization of behavioral data (i.e., the coding of graded outcomes relative to a validated cut‐off level regardless of its severity as either normal = 0 or abnormal = 1) which pulls together severe and mild deficits. This could be problematic as the latter could either reflect low levels of deficit in mildly affected patients or spatial biases in attentional orienting and alerting networks present in healthy, well‐adapted, normally‐functioning participants. In the current study, we used for each test the indicated outcome measures and cut‐off levels of normal/pathological performance established in the French guidelines of the GEREN battery. Although such occurrences were rare in our cohort, the use prior to binarization of left versus right ratios in cancellation tasks (such as the bells or the letter paradigms) may mask behind similar severity scores, very different clinical situations. Thus, future studies may need to capture symptom severity in a more precise manner, by considering the computation of additional outcome measures for spatially lateralized performance tests, preserving individual right and left performance (see Bartolomeo & Chokron, 1999 for a proposal), provided that these are well‐established and have validated cut‐off levels adapted to different populations.

Fourth, in the present work, our lesion‐based MSA study recruited neglect patients suffering unilateral right lesions, restricting our MSA analyses to ROIs in the right hemisphere. Nonetheless, influential anatomical models and experimental evidence emphasize a role for the left hemisphere in visuospatial deficits such as hemineglect (He et al., 2007; Malherbe et al., 2018, for an application of MSA to patients with left and right neglect following right and left hemisphere strokes, respectively). The contribution of left hemisphere ROIs to attentional orienting and the symptoms of neglect is also supported by studies showing increased radial diffusivity in parietal and bilateral occipital connections in this patient populations (Umarova et al., 2014, 2017) and the influence of left hemisphere regions determining clinical progress toward spontaneous recovery or an enduring deficit (Bartolomeo and Thiebaut de Schotten, 2016; Lunven et al., 2015).

Lastly, it could be argued that the MSA approach here employed lacked the ability to provide direct evidence of disconnection in the way and manner “disconnection syndromes” were originally defined and have been explored clinically. To overcome this challenging limitation, MSA studies should avoid cases with cortical damage and exclusively include patients with pure white matter damage, or alternatively, control for the white matter injury impinging into cortical gray matter areas. Additionally, the present study did not directly quantify or compared whether gray matter ROIs, or a combination of gray and white matter ROIs would better explain hemispatial neglect symptoms than white matter ROIs taken alone. Moreover, the disconnection index used to estimate the percentage of injured voxels for each white matter bundle in our coalition of players was computed on the basis of a probabilistic atlas of white matter tracts (Foulon et al., 2018). Many of the explored tracts are well‐established and have been identified in human brain dissections. Nonetheless, others lack proper ground truth in neuroimaging and the origin, termination or the existence of some of their branches remains debated. Therefore, whereas from a neuroscientific point of view our study addresses interactions between gray matter areas mediated by white matter bundles, from a “clinical disconnection” point of view, our ability to conclude remains limited.

### 4.4 Conclusions and future perspectives

In summary, our study presents the first MSA lesion analysis conducted exclusively on white matter tracts. Importantly, a prior MSA study in this same patient cohort identified the causal contributions of gray matter cortical sites, allowing us to address to which extent the contributions of gray and white matter to each neuropsychological test proved coherent. The consideration of these two studies sets the basis for an integrated anatomical model of brain regions subtending visuospatial attention and its associated dysfunctions—notably hemineglect—including cortical regions and white matter connections.

From a pragmatic perspective, results obtained in our study might contribute to identify and individualize interventional strategies which by using invasive and noninvasive brain stimulation technologies may rehabilitate neurological attentional deficits in brain damaged patients.

However, noninvasive brain stimulation approaches applied to the rehabilitation of the visuospatial attention disorders yielded conflicting results and instigate a debate concerning the mechanism of action of these techniques. Such a diversity of outcomes might also be explained by the multivariate nature of visuospatial attention disorders such as neglect. The recognition of subgroups on the basis of lesion configuration analysis may contribute in this regard, in as much as lesion analysis will be able to point to the specific mechanism underlying the expression of disabling manifestations in the individual patient. Multivariate approaches such as the MSA may play an important role in that direction, given their advantages relative to standard univariate methods of lesion‐symptom analysis. It is still difficult to define exactly how studies using crude behavioral measures (such as those reported in the present work) and how data reporting both positive and negative contributions of a structure to a behavior could benefit the field. This is a standing limitation put forward by our study that should be addressed by approaches based on sophisticated machine‐learning techniques able to play an important role in extracting essential features from the data, and consequently in the construction of rehabilitation strategies and model generation, integrating information from multiple sources.

## Data Availability

Data sharing is not applicable to this article as no new data were created or analyzed in this study.
